# Layered Management of Hypertrophic Scars and Keloids: From Silicone and Intralesional Triamcinolone Acetonide Plus 5-Fluorouracil to Adjuvant Strontium-90

**DOI:** 10.7759/cureus.101893

**Published:** 2026-01-20

**Authors:** Osama S Abbadi, Faris Abdon, Zuhoor S Othman, Khalid Abdel Aziz

**Affiliations:** 1 Biochemistry, Omdurman Islamic University, Omdurman, SDN; 2 Medical Sciences, Orotta College of Medicine and Health Sciences, Asmara, ERI; 3 General Surgery, Prince Osman Digna Referral Hospital, Port Sudan, SDN; 4 Plastic and Reconstructive Surgery, Al Qassimi Hospital, Sharjah, ARE

**Keywords:** 5-fluorouracil, cryotherapy, fractional carbon dioxide laser, hypertrophic scar, intralesional triamcinolone, keloid, silicone gel, strontium-90 brachytherapy

## Abstract

Hypertrophic scars and keloids are fibroproliferative scars that can cause disfigurement, pruritus, pain, contracture, and reduced quality of life. Outcomes remain inconsistent because phenotypes vary by anatomic site, thickness, skin type, and time since injury, and no single modality is uniformly curative. We performed a narrative review informed by a structured search of MEDLINE/PubMed, Embase, Cochrane Library, Web of Science, and Scopus (2000 to November 2025), including clinical trials, observational studies, systematic reviews, and consensus guidance with clinically actionable treatment parameters. Evidence most consistently supports early, low-risk modulation with silicone gel or sheets for the prevention and early treatment of hypertrophic scars. For established hypertrophic scars and keloids, intralesional triamcinolone acetonide combined with 5-fluorouracil provides a practical pharmacological backbone, offering more reliable flattening and symptom control than steroid monotherapy. Adjuncts should be selected according to dominant clinical features, including cryotherapy for relatively thin lesions, fractional carbon dioxide laser for thickness and texture, and botulinum toxin A when tension, pruritus, or contracture is prominent. For selected thin keloids, radiotherapy options include external beam techniques and brachytherapy; superficial beta-brachytherapy with strontium-90 may serve as consolidation after response to intralesional therapy or as targeted postoperative adjuvant at high-risk sites. Because pigmentary adverse effects are clinically important in darker skin types, procedural choices should incorporate explicit counseling and conservative parameters. A pragmatic algorithm, reassessment triggers, and a modality summary table are provided to support routine clinical decision-making.

## Introduction and background

Pathological skin scarring (principally hypertrophic scars (HS) and keloids) imposes aesthetic, functional, and psychosocial burdens. HS remains confined to the original wound and may remodel over time, whereas keloids typically extend beyond the wound edge and persist or recur after treatment; this clinicopathologic distinction guides prognosis and therapy [1-3]. Pruritus, pain, and contracture further reduce quality of life, especially when scars cross joints or visible areas [1,2].

Scar biology reflects persistent inflammation and dysregulated extracellular matrix (ECM) turnover with excessive collagen I/III, altered fibronectin and periostin, and reduced matrix modulators (e.g., decorin, dermatopontin), alongside aberrant fibroblast signaling in TGF-β/PI3K/Akt/mTOR and microRNA networks [2,4]. Transcriptional data implicate a wound-response signature (e.g., SERPINE1/PAI-1) that promotes myofibroblast activity and impaired remodeling [5]. In contrast, scarless fetal repair features higher hyaluronic acid, reduced inflammatory cytokine signaling, and a distinct MMP/TIMP balance-mechanistic clues that favor early hydration/occlusion and anti-inflammatory strategies [6]. These insights support pairing (pathway-targeted) therapies with approaches that modulate the mechanical and inflammatory microenvironment.

Clinically, no single modality is curative. Conventional options (silicone/compression, intralesional corticosteroids, 5-fluorouracil (5-FU), cryotherapy, lasers, surgery, and adjuvant radiotherapy) provide benefit but are limited by recurrence, side effects, and heterogeneity across phenotypes and sites [2,7]. Practical frameworks organize care into non-invasive, minimally invasive, and invasive strategies, emphasizing stepwise combinations tailored to lesion type, location, and symptoms [1,8].

Among non-invasive measures, topical silicone has randomized evidence for prophylaxis after surgery, improving scar symptoms and reducing HS/keloid formation compared with control [3]. When choosing format, a prospective comparison found similar efficacy between silicone gel and sheets on Vancouver Scar Scale (VSS) outcomes at one to three months, with greater convenience and adherence reported for gel-useful in mobile or exposed sites and warm climates [9]. Onion extract (*Allium cepa*) may improve color, but height reduction is driven by silicone; the combination achieved the best overall therapeutic index in a three-arm study and can be considered when erythema is prominent [10].

For established lesions, intralesional corticosteroids remain a cornerstone, but adding a second agent improves durability. Prospective and retrospective series support 5-FU alone or in combination with triamcinolone, with better flattening and symptom control and acceptable safety [11,12]. Meta-analytic syntheses similarly favor combinations over steroid monotherapy, and tension-modulating adjuncts are rational, given mechanotransduction in scar biology [13,14]. Cryotherapy helps reduce thickness but is limited by pain and pigmentary change, particularly in darker skin [7]. Energy-based devices (e.g., pulsed-dye and fractional CO_2_ lasers) can improve vascularity, pliability, and thickness, often as part of multimodal regimens [7,14]. Botulinum toxin-A (BTX-A) showed improvement in pediatric post-burn HS/keloids in an intra-patient randomized trial and is increasingly used to reduce tension and symptoms as an adjunct to intralesional or laser therapy [14,15]. Photodynamic therapy using indocyanine green (ICG-PDT) demonstrates fibroblast inhibition and early clinical signals; it is a plausible option for refractory disease within specialist pathways [16,17].

Surgery alone carries a high recurrence rate for classic keloids; combination strategies are preferred [8]. Adjuvant radiotherapy has evolved with a focus on timing and dose. Guidance emphasizes delivery within ~24 hours post-excision when indicated, with fractionation tailored to site and depth [8]. Randomized and prospective data show that strontium-90 (Sr-90; β-radiation) (as a superficial brachytherapy) can lower recurrence and stabilize ultrasound-measured thickness/elastic modulus, particularly for small, early keloids after response to intralesional therapy [18]. These findings support a pathway in which bulky or recurrent keloids undergo limited-margin excision followed by immediate adjuvant radiotherapy, with intralesional consolidation as needed [8,18]. Adjuvant radiotherapy is delivered using multiple techniques, most commonly external beam radiotherapy (EBRT) (including electron beam and X-ray-based approaches) or brachytherapy (interstitial or surface), depending on target depth and institutional resources [19,20]. In this review, Sr-90 is highlighted not as a universal radiotherapy solution, but because its highly superficial β-dose profile makes it a practical consolidation option for selected thin lesions within a layered treatment pathway [8,18].

Emerging multimodal protocols intentionally pair the pathway with the physics. A recent analysis reported that sequential BTX-A + fractional CO_2_ laser + topical growth factor outperformed monotherapies on thickness and validated scar scales without new safety signals, especially when started earlier after scar formation [14]. On the biologic front, topical formulations that down-regulate phosphorylated PI3K/Akt/mTOR reduced keloid size in experimental models, underscoring the translational potential of mTOR-axis modulation [21].

This review synthesizes established and emerging interventions for HS and keloids, grading their practical value (efficacy, durability, safety, and feasibility) and proposing a phenotype-aware, escalation-ready algorithm that integrates prevention, intralesional and energy-based combinations, and surgery with adjuvant radiotherapy where appropriate [1-3,5-15,18].

## Review

Methods

Search Strategy and Study Selection

We conducted a narrative review informed by a structured literature search, aimed at summarizing clinically actionable evidence and parameters for stepwise (layered) management of HS and keloids. We searched MEDLINE/PubMed, Embase, the Cochrane Library, Web of Science, and Scopus for English-language literature published from 2000 to November 2025 using combinations of terms related to HS/keloids and common interventions (e.g., silicone gel/sheets, intralesional corticosteroids, 5-FU, cryotherapy, lasers including fractional CO_2_, BTX-A, surgery, radiotherapy, and Sr-90). Reference lists of eligible studies and relevant guideline/consensus documents were manually screened to identify additional studies. This work was designed as a narrative review with structured searching and was not intended as a formal systematic review.

Eligibility and Data Handling

We included human studies addressing prevention and/or treatment of HS and keloids, including randomized trials, controlled studies, prospective/retrospective cohorts, and case series with clinically interpretable outcomes, as well as systematic reviews/meta-analyses and guideline/consensus statements. Translational/experimental studies were included when they directly informed therapeutic rationale or clinically used parameters. We excluded non-clinical papers without interpretable endpoints and studies lacking sufficient intervention detail to support practical implementation. From the included studies, we extracted key clinical information, including treatment modality, dose/parameters, treatment interval/session number when reported, follow-up duration, outcomes/recurrence definitions, and adverse effects.

Outcomes and Measures

Primary outcomes included scar severity and treatment response as reported using validated clinical measures and recurrence definitions used by the source studies. Where validated scales were used, the most common measures were the VSS and the Patient and Observer Scar Assessment Scale (POSAS), which capture domains such as vascularity/pigmentation, pliability, height/thickness, and patient-reported symptoms/appearance impact [22,23]. Objective adjunct outcomes (when reported) included ultrasound-based thickness and related biomechanical metrics, as well as symptom outcomes such as pruritus and pain.

Appraisal and Synthesis

Because interventions, dosing/parameters, follow-up periods, and outcome definitions varied substantially across studies, we did not attempt quantitative pooling. Instead, findings are presented as a structured narrative emphasizing: (1) first-line low-risk measures for prevention/early disease, (2) a practical pharmacologic backbone for established lesions (intralesional triamcinolone ± 5-FU), (3) phenotype- and site-informed adjuncts (e.g., cryotherapy, fractional CO_2_ laser, BTX-A), and (4) escalation/consolidation strategies including surgery with adjuvant radiotherapy and, in selected candidates, superficial β-brachytherapy using Sr-90.

Discussion

Management of HS and keloids remains challenging because biology and biomechanics conspire to sustain fibroproliferation long after the inciting injury has resolved. Across the studies reviewed, a coherent picture emerges: outcomes improve when care is layered, beginning with low-risk measures that modulate hydration and inflammation, moving to intralesional anti-proliferative therapy for established disease, and reserving procedures and adjuvant radiotherapy for selected phenotypes with a propensity to recur [1,2,7,8,13,18]. Key modalities, typical dosing parameters, expected outcomes or recurrence, and principal risks are summarized in Table 1 [3,7-18,24-29].

**Table 1:** Summary of management modalities for hypertrophic scars and keloids Notes: Doses/parameters are typically used and must be individualized according to the lesion site, thickness, and skin type. Evidence anchors (article/s) by row: Silicone: [3,9]; Onion extract ± silicone: [10]; Imiquimod: [17]; Compression: [24]; TAC: [13,25,26]; 5-FU ± TAC: [11-13,25,26]; BTX-A: [14,15]; Cryotherapy: [7,25,27]; Fractional CO_2_ ± BTX-A: [14]; ICG-PDT: [16,17]; Surgery + RT: [8]; Sr-90 consolidation: [18]; Mechanotherapy: [28,29] BTX-A: botulinum toxin type A; EBRT: external beam radiotherapy; GF: growth factor; HDR: high-dose rate; HS: hypertrophic scars; ICG-PDT: indocyanine green-mediated photodynamic therapy; LDR: low-dose rate; PIH: post-inflammatory hyperpigmentation; RCT: randomized controlled trial; Sr-90: strontium-90; TAC: triamcinolone acetonide; 5-FU: 5-fluorouracil; VSS: Vancouver Scar Scale

Modality	Best candidates/when to use	How it’s given (dose/parameters)	Typical outcomes/recurrence	Key risks
Silicone (gel or sheet)	First‑line prophylaxis and early HS; choose format for adherence (gel for mobile/visible/hot climates; sheets for flat/covered areas)	Thin film BID (gel) or near‑continuous wear (sheet) for ≥8-12 weeks	↓ erythema/itch/height vs control in RCT; gel ≈ sheet for efficacy; gel often easier to use	Contact dermatitis (rare); adherence is the main limiter
Onion extract (±silicone)	When color/erythema improvement is a priority, it is best as an adjunct to silicone	Topical gel once or twice daily; combine with silicone for the best overall effect	Improves color; limited effect on height/itch alone; combo outperforms either alone at 6 months	Mild irritation/odor
Imiquimod 5% (post-excision)	Select cases only; inconsistent evidence-avoid routine use	Nightly to incision for several weeks post‑excision (protocols vary)	Mixed/negative data for recurrence prevention after excision	Erythema/irritation; adherence issues
Compression/pressure therapy	Burn scars, where garments are feasible and supervised	20-30 mmHg garments for months	↓ collagen I/III; improved scar profile in cohorts	Discomfort, skin breakdown, and adherence challenges
TAC	Symptomatic/thick HS and keloids; often used as a backbone	10-40 mg/mL, q4-6 weeks; multiple sessions	↓ height/pruritus; variable recurrence; monotherapy less durable than combinations	Atrophy/telangiectasia; hypopigmentation
Intralesional 5-FU ± TAC (combo preferred)	Established, thicker lesions; steroid‑sparing or steroid‑resistant	5-FU 40-50 mg mixed with TAC (e.g., 10 mg/mL), weekly-biweekly cycles	Faster flattening and better durability than TAC alone across series; manageable local AEs	Pain/erosions; dose‑related ulceration
BTX-A	Prominent itch/contracture; high-tension sites; adjunct before laser or injections	Intradermal/intralesional mapping along tension lines; dose by lesion size	Improves VSS domains (pediatric RCT); helpful for pliability/symptoms	Injection pain; rare weakness if misplaced
Cryotherapy	Thinner, newer lesions; pigment-indifferent areas; as part of combination plans	Contact/spray techniques; multiple sessions (≥3)	Good–excellent responses in cohorts; symptom relief; plan for repeat sessions	Pain during freeze; hypopigmentation or PIH
Fractional CO₂ laser (±BTX-A, ±topical growth factor)	Textural/thick HS; earlier scars respond better; combine for greater gains	Device‑specific; low‑density fractional passes; consider sequence BTX‑A → CO_2_ → topical GF	Combo superior to monotherapy on VSS and thickness (retrospective 2024); generally safe	PIH, erythema, edema; consider test spot, conservative density, photoprotection
Photodynamic therapy (indocyanine green; ICG-PDT)	Refractory keloids, or when lasers/surgery are limited, are an adjunct in specialist care	Indocyanine-green + light source (protocols vary)	Suppresses fibroblast activity/collagen synthesis; early clinical signals	Photosensitivity, local reaction
Surgery + adjuvant radiotherapy	Bulky or recurrent keloids; plan adjuvant within 24 h post-excision	Excision/debulking → EBRT (electrons/orthovoltage/photons) or brachytherapy (HDR/LDR; source-dependent), site-dependent	Lower recurrence than surgery alone (site‑specific)	Radiation dermatitis; pigmentary change (PIH/hypopigmentation); logistics
Superficial β-brachytherapy with strontium-90 (Sr-90) after TAC + 5-FU	Small, relatively thin, responsive keloids where relapse risk is high	After serial TAC + 5‑FU when thinned to ~2-3 mm: 15-20 Gy in 3-4 daily fractions	Randomized data: ~half the 1‑yr recurrence vs injections alone; ultrasound shows stability	Local erythema/dryness; possible pigmentary change; careful shielding/collimation
Mechanotherapy/vacuum massage	Adjunct for pliability/symptom relief in specialist settings; not disease‑modifying alone	Intermittent negative‑pressure protocols; clinic‑based	Short‑term epidermal/dermal changes; clinical benefits uncertain	Bruising/discomfort; time/cost

Prevention and very early management should be adherence-friendly and straightforward. Randomized evidence supports topical silicone as a foundation therapy after closure, with reductions in erythema, pruritus, and early thickening versus control, and excellent acceptability [3]. When patients choose between formats, comparative data show similar efficacy for gels and sheets; gels are often preferred for mobile or visible sites and hot climates, while sheets suit flat, covered areas, so the practical choice is whichever format maximizes use rather than chasing a non-existent efficacy gap [9]. Onion extract adds value mainly for colour/erythema and works best when combined with silicone; by itself, it does little for height or itch [10]. Imiquimod 5% has been inconsistent after excision and should not be relied upon as a standalone prophylactic measure [17].

Once a scar is established and symptomatic, intralesional therapy becomes the workhorse. Corticosteroids alone retain utility, but converging prospective data indicate that adding 5-FU improves flattening and durability in many patients, with manageable local adverse effects that are mitigated by technique and dosing adjustments [11-13,25,26]. This combination also appears to limit steroid-related atrophy and telangiectasia compared with triamcinolone alone in some series [13,25,26]. BTX-A is an attractive adjunct when tension, pruritus, or surface rigidity dominate the phenotype; an intra-patient randomized pediatric trial demonstrated improvements across VSS domains with good tolerability, suggesting a role where symptom relief and pliability gains are priorities [30]. These findings align with the broader principle that reducing mechanical load and myofibroblast drive can amplify drug effects.

Energy-based procedures and photodynamic therapy can be sensibly integrated rather than used in isolation. Fractional CO_2_ laser improves texture and thickness and pairs well with anti-proliferative strategies. A contemporary multimodal sequence (BTX-A followed by fractional CO_2_ and a topical growth factor) outperformed monotherapies on validated scales and thickness, particularly when instituted relatively early [14]. ICG-PDT shows mechanistic and early clinical signals (suppression of keloid fibroblast activity and collagen synthesis), making it a plausible option in refractory disease where access to lasers or surgery is limited. However, controlled clinical trials are still needed [16,17]. Cryotherapy remains useful for thinner, newer lesions, with consistent symptom relief and flattening across cohorts; its trade-offs are pain and pigmentary change, which warrant careful counseling in darker skin types and thoughtful pairing with other modalities [7,25,27].

For keloids with significant bulk or a track record of relapse, surgery should rarely stand alone. Contemporary guidance emphasizes combination care and, when surgery is chosen, the importance of well-timed adjuvant radiotherapy to reduce recurrence, ideally within 24 hours of excision, depending on the site and modality [8]. Superficial β-brachytherapy with Sr-90 offers a pragmatic, targeted option in carefully selected non-surgical or post-injection scenarios. In a randomized setting, adjuvant Sr-90 delivered in short courses (e.g., 15-20 Gy over three to four days) approximately halved one-year recurrence compared with injections alone, not by further debulking but by stabilizing a good response; efficacy is most significant when lesions are pre-thinned to ~2-3 mm, so dose is deposited where it is needed [18]. This consolidation concept aligns with the broader theme of making early gains and then maintaining them.

Radiotherapy for keloid control can be delivered as EBRT (including electron beam radiotherapy and X-ray-based techniques) or as brachytherapy, where a source is positioned close to the target to achieve a steep dose gradient [19,20]. Clinical practice has used multiple radiotherapy modalities and sources, including electron beam radiotherapy, Ir-192 high-dose-rate (HDR) brachytherapy, and Sr-90 surface (plaque/dermal plate) brachytherapy, confirming that Sr-90 is one option within a broader radiotherapy toolkit [31,32]. We focus on Sr-90/Sr-90Y not to imply superiority over all radiotherapy techniques, but because it has a distinct niche in (layered) care as superficial consolidation: Sr-90 is a β-emitter, and its dose falls rapidly with depth to approximately 1-3% at 5 mm; about 75% of β-radiation is absorbed within the first 2 mm, with the remaining dose absorbed within the subsequent 1 mm of tissue [8]. This physical profile supports Sr-90 use when lesions are small and relatively thin, particularly after intralesional therapy has achieved meaningful thinning and symptom control, and the clinical objective becomes maintaining stability and reducing recurrence [8]. In a prospective, randomized, controlled trial, Sr-90 given after three triamcinolone acetonide (TAC) + 5-FU intralesional injections reduced the one-year recurrence rate from 85.7% to 44.4% in small, relatively young keloids, supporting its role as an adjuvant consolidation strategy rather than a primary debulking tool [18].

Technique selection should be guided by target depth and lesion geometry. Sr-90 is most appropriate when the intended target is superficial and thin, where rapid depth-dose fall-off is advantageous, and exposure to deeper tissues should be minimized [8]. For bulkier or deeper lesions, modalities that can prescribe dose at greater depths may be more appropriate, such as EBRT (commonly electrons or selected X-ray approaches) or interstitial HDR brachytherapy, where catheter placement and prescription depths typically aim to cover deeper target tissues (e.g., prescribed dose depth 4-7 mm has been reported for interstitial HDR after excision) [8,19]. Consistent with this, Ir-192 HDR brachytherapy has been used as an adjuvant approach in difficult recurrent cases, with protocols such as 15 Gy in three fractions reported in salvage settings [32]. Accordingly, Sr-90 should be presented as a selective superficial option within an overall radiotherapy toolkit rather than a universal substitute for EBRT or interstitial brachytherapy [8,19].

The biological rationale for these layered strategies is increasingly apparent. Aberrant signaling through TGF-β and PI3K/Akt/mTOR sustains collagen I/III deposition, alters fibronectin and periostin, and suppresses matrix modulators such as decorin and dermatopontin [2,4]. Transcriptional work highlights a wound-response program involving SERPINE1/PAI-1 that propagates myofibroblast activity and impaired remodeling [5]. In contrast, scarless fetal repair features higher hyaluronic acid levels, lower profibrotic cytokine tone, and a remodeling-favouring MMP/TIMP balance [6]. Interventions that hydrate/occlude, reduce inflammatory drive, down-shift fibroblast proliferation, and relieve mechanical tension therefore make mechanistic sense, and, in practice, combinations that (pair the pathway with the physics) consistently perform better than single agents [1,2,6,8,13,18,21].

Safety and practicality are central to real-world choices. Silicone is safe and inexpensive; adherence is the primary determinant of benefit [3,9]. Intralesional TAC + 5-FU typically requires weekly sessions early on; transient pain and superficial erosions are common trade-offs that can be reduced with smaller aliquots, the fanning technique, and spacing [11,12]. Cryotherapy and lasers carry a risk of post-inflammatory hyperpigmentation (PIH); therefore, parameter selection and sun protection are essential, particularly in individuals with darker skin types [7,27,33-35]. With Sr-90, careful patient selection and shielding are needed; reported acute toxicity is low when lesions are thin, and fields are well collimated [18]. BTX-A has been well tolerated in both pediatric randomized studies and adult series, and its effect on tension likely complements intralesional and laser-based treatment plans [13,30].

Considerations in Darker Skin Types and Pigmentary Change Risk

Keloids disproportionately affect individuals with more pigmented skin (Fitzpatrick skin types IV-VI), and pigmentary safety and counseling are therefore central to real-world management [36,37].

Pigmentary adverse effects are particularly relevant when using cryotherapy and energy-based devices. In the keloid cryotherapy series, hypopigmentation is a recognized adverse effect [7,33-35,38]. Fractional laser procedures in higher Fitzpatrick phototypes also carry a risk of PIH [7,33-35,38].

Practical risk-reduction measures include explicit expectation setting, baseline photography, conservative parameter selection, considering test spots when feasible, strict photoprotection, and close follow-up to detect and manage pigmentary change early [34,35].

When adjuvant radiotherapy or brachytherapy is used as consolidation or post-excision control, patients should also be counseled that pigmentary change and telangiectasia can occur, even though acute reactions are typically mild and self-limited [18-20,31,32].

**Figure 1:**
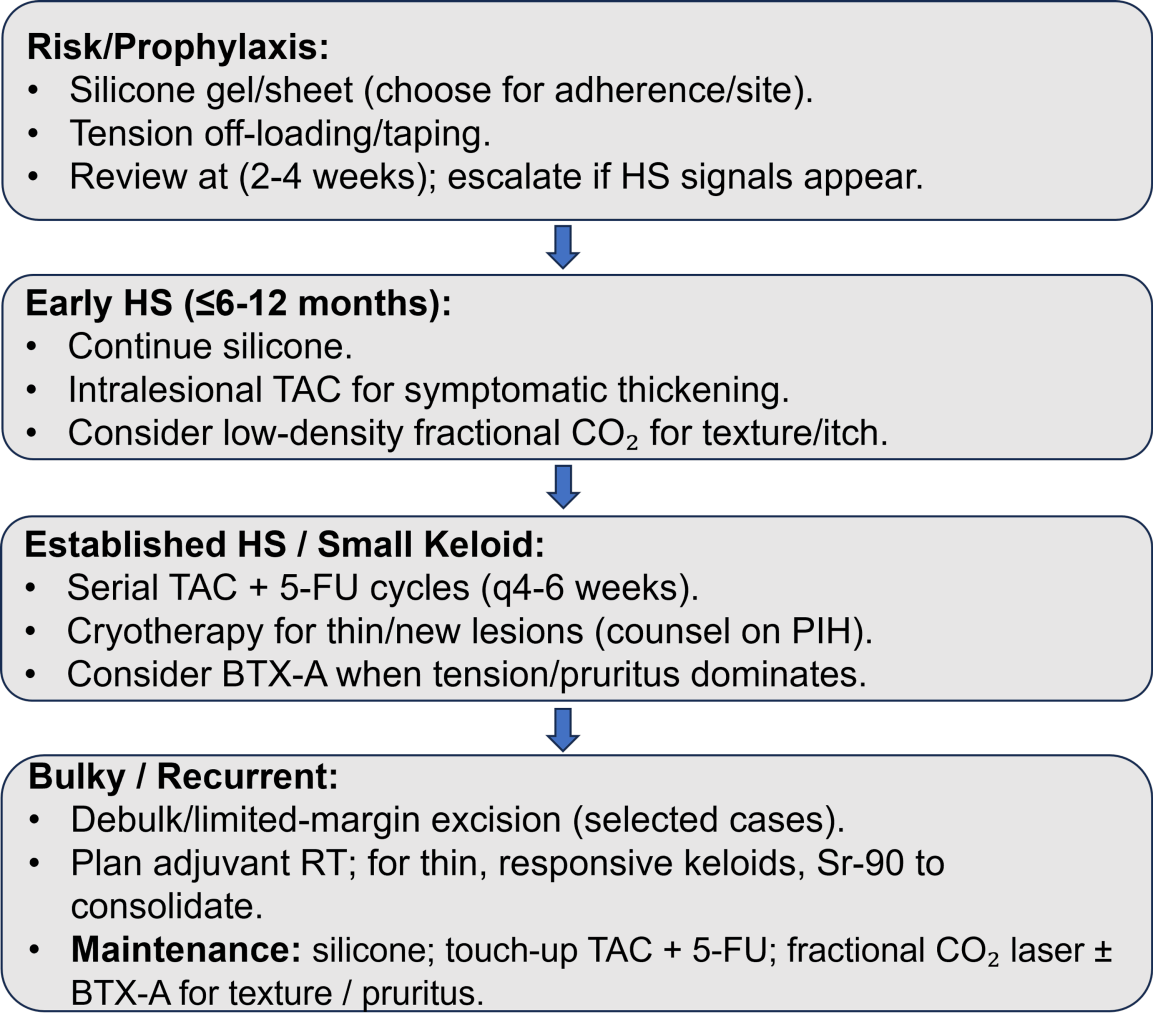
Clinical algorithm for layered management of hypertrophic scars and keloids Early measures emphasize hydration and tension reduction (silicone as first-line treatment); TAC + 5-FU is the pharmacologic backbone for established disease, with cryotherapy, fractional CO_2_, and BTX-A added based on phenotype. In selected, relatively thin keloids, Sr‑90 can consolidate response or follow excision at high‑risk sites. Sources (article/s): silicone [3,9]; TAC + 5‑FU [11-13,25,26]; cryotherapy [7,25,27]; BTX‑A and multimodal sequences [14,15]; fractional CO_2_ combinations [14]; adjuvant radiotherapy/Sr‑90 [8,18]; adjuncts (onion extract [10]; imiquimod negative [17]). HS: hypertrophic scars; TAC: triamcinolone acetonide; 5-FU: 5-fluorouracil; BTX-A: botulinum toxin type A; Sr-90: strontium-90; RT: radiotherapy

Our phenotype-aware, layered approach is presented in Figure 1 (clinical treatment algorithm) [3,7-15,17,18,25-27], which maps prevention/early modulation (silicone; tension off-loading), the intralesional backbone (TAC + 5-FU), escalation options (cryotherapy, fractional CO_2_, BTX-A), and consolidation with Sr-90 in selected cases.

The algorithm is intended as a pragmatic escalation pathway rather than a rigid protocol, and site, thickness, symptoms, skin type, and tolerance should be used to individualize reassessment intervals. As a practical guide, early noninvasive therapy with silicone can be reassessed at about 8-12 weeks (consistent with common trial follow-up structures) to confirm symptomatic improvement and early flattening and to address adherence [3,9] For established lesions treated with intralesional corticosteroids, a response check after approximately three sessions given every four to six weeks is a reasonable trigger to either continue the same regimen or add/transition to combination therapy when improvement is clinically small or plateaus [13,25,26,39]. When 5-FU-based injection regimens are used weekly to biweekly, reassessment after roughly four to six sessions is typically sufficient to judge whether meaningful flattening and symptom control are progressing [26].

For procedural adjuncts, both cryotherapy and fractional laser protocols are commonly delivered as multi-session courses; therefore, a minimum trial of about three sessions is a practical benchmark before declaring non-response, unless adverse effects, intolerance, or pigmentary risk necessitate earlier modification [7,27,38,40]. Across modalities, escalation should be accelerated when there is functional impairment (e.g., contracture), rapid progression, early relapse after an initial response, recurrent ulceration, or unacceptable adverse effects with the current modality [13,25,26,39].

Taken together, the evidence supports a pragmatic, phenotype-aware approach. After surgery or trauma at high-risk sites, begin silicone promptly and consider tension-offloading; move to intralesional TAC + 5-FU if hypertrophy develops despite early measures. For small, relatively thin primary keloids, TAC + 5-FU, with or without cryotherapy, is a reasonable first step; if responses are good but short-lived, consolidation with Sr-90 can extend remission [7,18]. Bulky or recurrent lesions fare better with staged debulking or limited-margin excision embedded in a program that plans adjuvant radiotherapy from the outset, rather than as a last resort [8]. Across scenarios where tension, pruritus, or stiffness predominate, BTX-A and fractional CO_2_ laser (sometimes in sequence with topical growth factors) are logical additions [30]. Photodynamic therapy can be considered in specialist pathways for refractory disease or when resource constraints limit other options [16,17]. Onion extract is best reserved as a color-directed adjunct alongside silicone [10].

The literature remains heterogeneous. Definitions of recurrence vary, follow-up is often brief, and many studies are single-center with modest sample sizes. Mechanotherapy (vacuum massage/negative-pressure massage) shows measurable, short-term structural changes and plausible mechanotransduction effects, but clinical endpoints are inconsistent, and studies are small with risk of bias; at present, it is best viewed as an adjunct for pliability and comfort within a broader plan rather than a disease-modifying therapy [28,29]. These limitations suggest conservative, combination-oriented recommendations and emphasize the need for standardization. Priorities for future work include harmonized outcome sets that pair POSAS/VSS with objective ultrasound or elastography, consensus definitions and timepoints for recurrence (at least 12 months), and pragmatic comparisons of common sequences (e.g., cryotherapy followed by TAC + 5-FU then an adjuvant modality) versus monotherapies. Mechanism-guided trials (targeting PI3K/Akt/mTOR or related nodes and testing ICG-PDT in controlled designs) are also warranted [5,16,17,21].

In summary, durable control of HS and keloids is most likely when clinicians match treatment to phenotype, start early with adherence-friendly measures, deploy intralesional combinations as a backbone, and stabilize good responses with procedures or adjuvant radiotherapy where appropriate. This strategy is supported by randomized and prospective data for silicone, BTX-A, TAC + 5-FU, and Sr-90 in defined contexts, anchored by an increasingly coherent mechanistic rationale [3,8-13,18,30,41,42].

## Conclusions

A single therapy rarely controls HS and keloids. The most consistent gains come from layered care: begin with prevention and early modulation (silicone; tension control), anchor treatment of established lesions with intralesional TAC + 5-FU, and add phenotype-specific options (cryo, fractional CO_2_, BTX-A) to address thickness, texture, pruritus, and tension. In selected, relatively thin keloids that respond to injections (or after excision at high-risk sites), adjuvant radiotherapy, including superficial beta-brachytherapy with Sr-90, can reduce recurrence. In darker skin types, management should explicitly address pigmentary risk when selecting cryotherapy, lasers, or radiotherapy. This framework balances efficacy, safety, and feasibility and can be adapted to lesion site, patient preferences, and resource availability.

## References

[1] Rabello FB, Souza CD, Farina Júnior JA (2014). Update on hypertrophic scar treatment. Clinics (Sao Paulo).

[2] Bayat A, McGrouther DA, Ferguson MW (2003). Skin scarring. BMJ.

[3] de Giorgi V, Sestini S, Mannone F, Papi F, Alfaioli B, Gori A, Lotti T (2009). The use of silicone gel in the treatment of fresh surgical scars: a randomized study. Clin Exp Dermatol.

[4] Ud-Din S, Volk SW, Bayat A (2014). Regenerative healing, scar-free healing and scar formation across the species: current concepts and future perspectives. Exp Dermatol.

[5] Peake MA, Caley M, Giles PJ (2014). Identification of a transcriptional signature for the wound healing continuum. Wound Repair Regen.

[6] Colwell AS, Longaker MT, Lorenz HP (2003). Fetal wound healing. Front Biosci.

[7] Barara M, Mendiratta V, Chander R (2012). Cryotherapy in treatment of keloids: evaluation of factors affecting treatment outcome. J Cutan Aesthet Surg.

[8] Nien HH, Yu PC, Yen YH, Tsai YL, Wang LY, Hsieh CH (2025). Radiotherapy for keloids: a comprehensive narrative review. Cureus.

[9] Kim SM, Choi JS, Lee JH, Kim YJ, Jun YJ (2014). Prevention of postsurgical scars: comparsion of efficacy and convenience between silicone gel sheet and topical silicone gel. J Korean Med Sci.

[10] Hosnuter M, Payasli C, Isikdemir A, Tekerekoglu B (2007). The effects of onion extract on hypertrophic and keloid scars. J Wound Care.

[11] Fitzpatrick RE (1999). Treatment of inflamed hypertrophic scars using intralesional 5-FU. Dermatol Surg.

[12] Nanda S, Reddy BS (2004). Intralesional 5-fluorouracil as a treatment modality of keloids. Dermatol Surg.

[13] Sutedja EK, Sutedja E, Ruchiatan K, Faldian Y, Ismiranty D, Putri AD (2025). Intralesional botulinum toxin A for keloid treatment: a review of efficacy, safety, and clinical applications. Clin Cosmet Investig Dermatol.

[14] Wang J, Huang L, Li J (2024). Efficacy and safety of sequential treatment with botulinum toxin type A, fractional CO2 laser, and topical growth factor for hypertrophic scar management: a retrospective analysis. Sci Rep.

[15] Goel A, Shrivastava P (2010). Post-burn scars and scar contractures. Indian J Plast Surg.

[16] Shao J, Hu M, Wang W (2024). Indocyanine green based photodynamic therapy for keloids: fundamental investigation and clinical improvement. Photodiagnosis Photodyn Ther.

[17] Cação FM, Tanaka V, Messina MC (2009). Failure of imiquimod 5% cream to prevent recurrence of surgically excised trunk keloids. Dermatol Surg.

[18] Deng K, Xiao H, Liu X, Ogawa R, Xu X, Liu Y (2021). Strontium-90 brachytherapy following intralesional triamcinolone and 5-fluorouracil injections for keloid treatment: a randomized controlled trial. PLoS One.

[19] Liu EK, Cohen RF, Chiu ES (2022). Radiation therapy modalities for keloid management: a critical review. J Plast Reconstr Aesthet Surg.

[20] Xu J, Yang E, Yu NZ, Long X (2017). Radiation therapy in keloids treatment: history, strategy, effectiveness, and complication. Chin Med J (Engl).

[21] Tang Z, Cao Y, Ding J, Zhai X, Jing M, Wang M, Lu L (2020). Wubeizi ointment suppresses keloid formation through modulation of the mTOR pathway. Biomed Res Int.

[22] Sullivan T, Smith J, Kermode J, McIver E, Courtemanche DJ (1990). Rating the burn scar. J Burn Care Rehabil.

[23] Draaijers LJ, Tempelman FR, Botman YA, Tuinebreijer WE, Middelkoop E, Kreis RW, van Zuijlen PP (2004). The patient and observer scar assessment scale: a reliable and feasible tool for scar evaluation. Plast Reconstr Surg.

[24] Kwon SY, Park SD, Park K (2014). Comparative effect of topical silicone gel and topical tretinoin cream for the prevention of hypertrophic scar and keloid formation and the improvement of scars. J Eur Acad Dermatol Venereol.

[25] Gupta S, Sharma VK (2011). Standard guidelines of care: keloids and hypertrophic scars. Indian J Dermatol Venereol Leprol.

[26] Shah VV, Aldahan AS, Mlacker S, Alsaidan M, Samarkandy S, Nouri K (2016). 5-fluorouracil in the treatment of keloids and hypertrophic scars: a comprehensive review of the literature. Dermatol Ther (Heidelb).

[27] Zouboulis CC, Blume U, Büttner P, Orfanos CE (1993). Outcomes of cryosurgery in keloids and hypertrophic scars. A prospective consecutive trial of case series. Arch Dermatol.

[28] Meirte J, Moortgat P, Anthonissen M (2016). Short-term effects of vacuum massage on epidermal and dermal thickness and density in burn scars: an experimental study. Burns Trauma.

[29] Moortgat P, Anthonissen M, Meirte J, Van Daele U, Maertens K (2016). The physical and physiological effects of vacuum massage on the different skin layers: a current status of the literature. Burns Trauma.

[30] Tawfik AA, Ali RA (2023). Evaluation of botulinum toxin type A for treating post burn hypertrophic scars and keloid in children: an intra-patient randomized controlled study. J Cosmet Dermatol.

[31] Lee SY, Park J (2015). Postoperative electron beam radiotherapy for keloids: treatment outcome and factors associated with occurrence and recurrence. Ann Dermatol.

[32] Garg MK, Weiss P, Sharma AK (2004). Adjuvant high dose rate brachytherapy (Ir-192) in the management of keloids which have recurred after surgical excision and external radiation. Radiother Oncol.

[33] Clark CM, Silverberg JI, Alexis AF (2013). A retrospective chart review to assess the safety of nonablative fractional laser resurfacing in Fitzpatrick skin types IV to VI. J Drugs Dermatol.

[34] Kaushik SB, Alexis AF (2017). Nonablative fractional laser resurfacing in skin of color: evidence-based review. J Clin Aesthet Dermatol.

[35] Sequeira Campos MB, Xiao A, Ettefagh L (2025). Laser revision of scars. StatPearls [Internet].

[36] Tchero H (2020). Management of scars in skin of color. Textbook on Scar Management: State of the Art Management and Emerging Technologies.

[37] Yedomon GH, Adegbidi H, Atadokpede F, Akpadjan F, Mouto EJ, do Ango-Padonou F (2012). Keloids on dark skin: a consecutive series of 456 cases [Article in French]. Med Sante Trop.

[38] Rusciani L, Rossi G, Bono R (1993). Use of cryotherapy in the treatment of keloids. J Dermatol Surg Oncol.

[39] Gold MH, McGuire M, Mustoe TA, Pusic A, Sachdev M, Waibel J, Murcia C (2014). Updated international clinical recommendations on scar management: part 2 - algorithms for scar prevention and treatment. Dermatol Surg.

[40] Tawfic SO, El-Tawdy A, Shalaby S, Foad A, Shaker O, Sayed SS, Metwally D (2020). Evaluation of fractional CO(2) versus long pulsed Nd:YAG lasers in treatment of hypertrophic scars and keloids: a randomized clinical trial. Lasers Surg Med.

[41] Apikian M, Goodman G (2004). Intralesional 5-fluorouracil in the treatment of keloid scars. Australas J Dermatol.

[42] Raslan EE, Bakhamees BH, Howsawi TA (2024). The efficacy of botulinum toxin type A (BTA) in the treatment of hypertrophic scars and keloids: a systematic review and meta-analysis of randomized controlled trials. Cureus.

